# Muscle co-activation in the elderly contributes to control of hip and knee joint torque and endpoint force

**DOI:** 10.1038/s41598-023-34208-6

**Published:** 2023-05-02

**Authors:** Keisuke Kubota, Moeka Yokoyama, Hiroki Hanawa, Taku Miyazawa, Keisuke Hirata, Katsuya Onitsuka, Tsutomu Fujino, Naohiko Kanemura

**Affiliations:** 1 Research Development Center, Saitama Prefectural University, Saitama, Japan; 2 Sportology Center, Graduate School of Medicine, Juntendo University, Tokyo, Japan; 3 Department of Rehabilitation, Faculty of Health Sciences, University of Human Arts and Science, Saitama, Japan; 4 Department of Rehabilitation, Faculty of Health Sciences, Tokyo Kasei University, Saitama, Japan; 5 Graduate Course of Health and Social Services, Saitama Prefectural University, 820 Sannomiya, Koshigaya, Saitama, 343-8540 Japan

**Keywords:** Neurophysiology, Motor control, Ageing

## Abstract

We investigated the coordinated activity patterns of muscles based on cosine tuning in the elderly during an isometric force exertion task. We also clarified whether these coordinated activity patterns contribute to the control of hip and knee joint torque and endpoint force as co-activation. Preferred direction (PD) of activity for each muscle in 10 young and 8 older males was calculated from the lower limb muscle activity during isometric force exertion task in various directions. The covariance of endpoint force (η) was calculated from the exerted force data using a force sensor. Relationship between PD and η was used to examine the effect of muscle co-activation on the control of endpoint force. Co-activation between rectus femoris and semitendinosus/biceps femoris increased with changes in muscle PD. Additionally, the η values were significantly low, suggesting that co-activation of multiple muscles may contribute to endpoint force exertion. The mechanism for cooperative muscle activity is determined by the cosine tuning of the PD of each muscle, which affects the generation of hip and knee joint torque and endpoint force exertion. Co-activation of each muscle’s PD changes with age, causing increased muscle co-activation to control torque and force. We demonstrated that co-activation in the elderly is a stabilizer of unsteady joints and a muscle control strategy for cooperative muscle activity.

## Introduction

Aging is associated with irreversible degenerative changes that markedly reduce neuromuscular function. The characteristic neuromuscular functional changes associated with aging involve morphological muscle degeneration, such as reduction in muscle mass^1^ and muscle physiological cross-sectional area^2^. Moreover, ineffective descending motor command may lead to impaired motor unit recruitment and reduced discharge rate in older age^3,4^. These impairments may result in ineffective mechanical output to reach the desired force exertion level^3^. Motor unit activation may also become ineffective with advanced age due to an altered balance between the excitation and inhibition of spinal circuits^5^. In these cases, the muscle activity pattern associated with joint movement may differ from that of young people, although they exert the same force. Therefore, understanding the general pattern of multiple muscles in coordinated activity during force exertion in the elderly is important to elucidate age-related changes in motor control.

The characteristic muscle activity pattern in the elderly is represented by the co-activation of muscles, which is defined as the synchronized activities of agonist and antagonist muscles^6^. The co-activation of agonist and antagonist muscles is important for task-learning processes^7^ or joint stability^8^. Moreover, muscle co-activation increases with age^9^ and contributes to improved joint stability; however, it also increases fall risk^10^ and energy metabolism^9,11^. Additionally, the progression of these abnormalities may lead to pathological conditions, such as osteoarthritis^12,13^. Thus, excessive co-activation in the elderly is detrimental.

The pattern of coordinated activity of multiple muscles, such as co-activation, is also involved in the exertion of force at the endpoint via joint torque. Kumamoto et al.^14^ reported that the existence of an antagonistic pair of bi-articular muscles on the upper leg is necessary for precise control of the position and force exerted on the hand. In fact, the rectus femoris (RF) and hamstrings are active during phases II and III, in which the center of body mass is lifted upward during sit-to-stand movements^15,16^. This co-activation between the agonist (hamstrings) and antagonist (rectus femoris [RF]) muscles may be interpreted as a strategy to increase joint stability. However, it may also be a strategy to control the direction of the exerted force. Regardless, it is unclear how co-activation in the elderly contributes to endpoint force.

This contribution may be determined by assessing (1) the preferred direction (PD) in which each muscle is likely to exert force and (2) the variability of the endpoint force. The direction of force exertion at the endpoint depends on the combination of the involved joint torques. Furthermore, torque combination is generated by the coordinated activity of different muscles. Muscle PD represents the direction in which a given muscle is most likely to be active when two adjacent joint torques are generated simultaneously in various magnitudes and combinations. The desired torque vector is exerted by the coordinated activity of each muscle, as determined by its positive projection from the PD (cosine tuning)^17^. It has been demonstrated that the characteristic distribution of each muscle’s PD is the result of optimization by the sum of the squared muscle activity of all muscles involved in the task^17–19^. Such muscle coordination activity pattern based on the cosine function of each muscle PD generates a joint torque, which in turn contributes to the endpoint force output.

Moreover, motor output includes the desired endpoint force and fluctuations in the presence of signal-dependent noise^20,21^. Kutch et al.^22^ found that the activation of any single muscle, as a prime mover, generates an average force vector in endpoint force space, with force covariance due to signal-dependent noise. Thus, the variability consistent with the direction in which the endpoint force is exerted (covariance) can be determined as the coordinated activity of many muscles to control the endpoint force, even in the case of muscle co-activity. Mapping PD-based muscle coordination activity patterns and covariance of the endpoint force help elucidate the mechanisms of coordination of multiple muscles in the elderly when isometrically challenged to exert force in all directions.

Therefore, in this study, we investigated how the coordinated activity patterns of muscles change in the elderly during an isometric force exertion task, based on cosine tuning. We also clarified whether these coordinated activity patterns contribute to the control of hip and knee joint torque and endpoint force as co-activation. Our hypothesis is that each muscle PD in the elderly may be more likely to be co-activated for endpoint exertion by deviating closer to each other. In addition, the covariance of the endpoint is expected to be low because of this co-activity. Research on muscle co-activation in the elderly, which has been considered a strategy to enhance joint stability, may provide new insights into its importance as a coordinated activity for endpoint force exertion.

## Materials and methods

### Participants

Ten young males (Y group) (mean ± standard deviation [SD]; age: 21 ± 1 years, height: 1.72 ± 0.03 m, body weight: 65.3 ± 3.7 kg) and eight elderly males (E group) (mean ± SD; age: 68 ± 1 years, height: 1.71 ± 0.03 m, body weight: 68.6 ± 3.0 kg) volunteered to participate in this study. Exclusion criteria were a history of central nervous system disease, trauma or surgery that would alter gait, or a history of serious cardiac or pulmonary disease. In addition to the aforementioned medical history, medical screening^23^ was conducted to verbally confirm the presence or absence of joint pain and medications and to ensure the absence of central nervous system, neuromuscular, or musculoskeletal disease. The experiments were explained in detail, and written informed consent was obtained from all participants. All procedures used in this study followed the Declaration of Helsinki and were approved by the ethics review committee of Saitama Prefectural University (19519).

### Protocol

The participants were placed in a left lateral supine position, with 90° hip and knee flexion of the right lower limb. The right lower limb was suspended in a sling parallel to the floor (Fig. S1a and b). The right ankle joint was attached to a vertical rail fixed to the wall using an attachment. The participants performed a task involving force exertion in various directions, as shown in Fig. S1c. A 6-axis force sensor (055YA501, Leptrino, Nagano, Japan) was mounted to the attachment piece and the force exerted on the ankle joint (F_x_, F_y_) was measured. The participant was instructed to maintain 50 N of force at the ankle joint^24^ for approximately 6 s^17^ in a specific direction. The direction of the endpoint force exertion was 12 directions in the sagittal plane (30°, 60°, 90°, 120°, 150°, 180°, 210°, 240°, 270°, 300°, 330°, and 360°)^25,26^. To ensure that the participant was able to exert force accurately in each direction, practice was conducted prior to the measurement. The measurement was undertaken three times for each direction with a total of 36 trials per participant (12 directions × 3 repetitions). One set consisted of 12 directions. At least 1 min of rest was taken between sets to minimize muscle fatigue. The direction and magnitude of the endpoint force exerted by the participant and target value (50 N) in each direction were displayed in real-time as red and black dots, respectively, on a monitor placed in front of the participants (Fig. S1d). The participant was instructed to bring the red dot close to the black dot and hold it there for 6 s.

### Data collection

Surface electromyography (EMG) data were collected at 1000 Hz using a wireless EMG system (Delsys Trigno Wireless System, DELSYS, Massachusetts, USA). Six right lower limb muscles were measured: gluteus maximus (GM), RF, vastus medialis (VM), vastus lateralis (VL), semitendinosus (ST), and biceps femoris (BF). The position of the electrode on each muscle followed the criteria for noninvasive evaluation by surface EMG (Surface Electromyography for the Non-Invasive Assessment of Muscle: SENIAM)^27^. The influence of skin noise was minimized by removing sebum using cotton saturated with alcohol before applying the electrodes. The endpoint forces (F_x_ and F_y_) were measured at 1000 Hz using a 6-axis force sensor. All data were synchronized using Vicon Workstation software.

### Calculation of the hip and knee joint torque

The hip and knee joint torque data, T = (T_k_, T_h_), were transformed from the endpoint force vector, F = (F_x_, F_y_). The conversion equation from F to T is expressed as follows:

1 $$\begin{array}{*{20}c} {T^{T} = AF^{T} ,} \\ \end{array}$$

where ^T^ represents transposition, and *A* denotes the moment arm matrix.

2 $$\begin{array}{*{20}c} {A = \left( {\begin{array}{*{20}c} {l_{1} } & 0 \\ {l_{1} + l_{2} \cos_{\varphi } } & {l_{2} \sin_{\varphi } } \\ \end{array} } \right),} \\ \end{array}$$

where l_1_ is the length of the thigh (from the greater trochanter to the lateral epicondyle), and l_2_ is the length of the shank (from the lateral epicondyle to the external capsule) (Fig. S2).

### Calculation of the PD

An EMG was acquired during 4 s of stable force out of 6 s of the measured force. The root mean squared value of the EMG activity during this period was used to evaluate the muscle activation level. The PD of each muscle was calculated using the following method. Multiple linear regression analysis with three variables was used to create a regression plane explaining the distribution of the muscle activity levels (Fig. S3) modeled in Eq. (3).

3 $$\begin{array}{*{20}c} {EMG = aT_{h} + bT_{k} ,} \\ \end{array}$$

where *a* and *b* are the regression coefficients of the hip and knee joint torques, respectively. The PD was calculated using Eq. (4) for the regression coefficients (*a*, *b*).

4 $$\begin{array}{*{20}c} {PD = \tan^{ - 1} \frac{b}{a}} \\ \end{array}$$

### Calculation of the endpoint force covariance

The covariance of the endpoint force when exerting a force in each direction was evaluated using the covariance of endpoint force (*η*) in Eq. (5)^20,22,28^. The endpoint force was evaluated for 4 s of stable force out of 6 s of the measured force. To reduce the voluntary contribution to the force variability, each force (F_x_, F_y_) was filtered using a 5–30 Hz Butterworth bandpass filter^22^ (Fig. S4a and b).

5 $$\begin{array}{*{20}c} {\eta = \frac{{\hat{F}_{target}^{T} {\text{cov}} \left[ {\tilde{F}} \right]\hat{F}_{target} }}{{Trace\left\{ {{\text{cov}} \left[ {\tilde{F}} \right]} \right\}}},} \\ \end{array}$$

where the numerator quantifies the amount of variance in the force exerted in the target direction, and the denominator summarizes the total amount of variance as a scalar. The F_target_ is defined as being equal to the average force vector $\overline{F}$, and ${\hat{\text{F}}}_{{{\text{target}}}}$ is defined as the unit vector in that direction. The $cov\left[ {{\tilde{\text{F}}}} \right]$ is the covariance matrix calculated from the filtered F_x_ and F_y_.

As shown in Fig. S4d, if the covariance of the endpoint force is consistent with the target direction and elliptical, then *η* is 1. However, if the endpoint force does not coincide with the target and shows a circular orthogonal ellipse, then *η* approaches 0.

### Quantification of RF-ST and RF-BF overlapping range

The proximity of each muscle PD suggests that these muscles contribute highly to force exertion in that direction. A previous study reported that a range of PD ± 90° was defined as the active range of the muscle and that an overlap of this range implied muscle co-activation^17^. In this study, we determined whether the coordinated activity patterns contribute to endpoint force as co-activation. Therefore, we quantified the extent to which the muscles were co-activated by calculating the overlapping activity range of RF-ST and RF-BF, which originally acted antagonistically.

### Calculation of the co-contraction of each muscle

To strengthen the result of co-activation in the overlapping range of PD ± 90° and explain the changes in η by muscle co-activation, the co-contraction index (CCI) was used for evaluation. For EMG normalization, the peak dynamic method was chosen, which normalizes by the maximum value of activity during a task^29,30^. This method does not require measuring the maximal voluntary contraction of muscle. Therefore, it minimizes muscle fatigue in the elderly. In this study, the amplitude of each muscle activity was normalized by the maximum value among the 36 trials (12 directions × 3 times). EMGs for the three trials in each direction were averaged as one representative value in each direction. The following formula (6) was used to calculate CCI:

6 $$\begin{array}{*{20}c} {CCI = \left( {EMG_{L} + EMG_{M} } \right) \times \frac{{EMG_{L} }}{{EMG_{M} }},} \\ \end{array}$$

where EMG_L_ is the activity level of the less active muscle, and EMG_M_ is the activity level of the more active muscle^31^. The amplitude-normalized EMG is expressed in the range of 0–1. Therefore, if both target muscles show a maximum value of 1, the CCI may be 2 at the most. In other words, if the two muscles simultaneously contracted completely, the CCI would be 2; meanwhile, if they did not contract simultaneously, the CCI would be 0. In this study, CCI was calculated for all one-to-one combinations of all muscles, totaling 15 combinations: RF-ST, RF-BF, RF-GM, RF-VM, RF-VL, GM-ST, GM-BF, GM-VM, GM-VL, VM-ST, VM-BF, VM-VL, VL-ST, VL-BF, and ST-BF.

### Statistical analysis

The covariance of the endpoint force was compared between directions using one-way analysis of variance (ANOVA) because the Shapiro–Wilk test indicated normality of the data. If a significant difference was found in one-way ANOVA, Tukey–Kramer test for post-hoc multiple comparisons was performed to determine the relationships between directions. Next, comparisons between Y group and E group were made using the following procedure. We verified the normality of the data by the Shapiro–Wilk test. If all data were significant at *P* < 0.05, we considered them non-normally distributed and calculated using the Wilcoxon rank sum test. Otherwise, the unpaired t-test was used. Accordingly, the unpaired t-test was used for the η of each direction and Wilcoxon rank sum test for the PD value, overlapping range of PD ± 90°, and CCI between Y group and E group. Statistical significance was set at *P* < 0.05.

## Results

**Figure 1:**
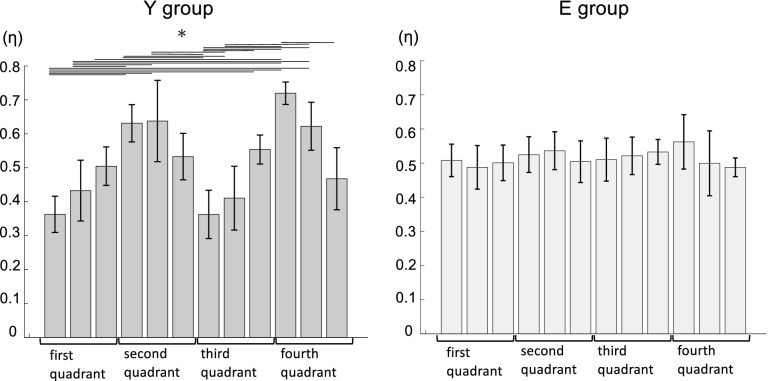
Comparison of the covariance of endpoint force (η) ± standard deviation in the 12 directions (D1–12) of the endpoint of force exertion using ANOVA; Y group (left) and E group (right). Each horizontal line indicates the direction in which a significant difference was found via post-hoc multiple comparisons. *P-values < 0.05.

**Figure 2:**
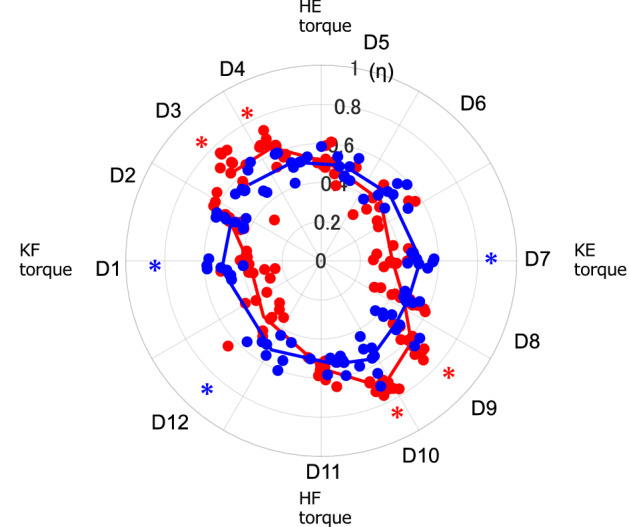
Circular plot of the covariance of endpoint force (*η*) of Y group (red) and E group (blue) in the torque plane. The dots represent the *η* value in each direction for all tested participants, while the lines depict the average values in the 12 directions of the force exertion endpoint. The red asterisks indicate the direction in which Y group is significantly higher, while the blue asterisks the direction in which E group is significantly higher. KE; knee extension, KF; knee flexion, HE; hip extension, HF; hip flexion.

Figure 1 shows the results of the covariance of endpoint force (η). The η values were significantly higher in the second (hip extension and knee flexion torque) and fourth (hip flexion and knee extension torque) quadrants of the hip and knee joint torque plane than in the first (hip and knee extension torque) and third (hip and knee flexion torque) quadrants in Y group (*P* < 0.001). However, there was no significant difference in the η values for E group in either quadrant. Figure 2 shows the result of the comparison of the η values. The η values in the second and fourth quadrants in the torque plane were significantly higher in Y group than in E group (*P* < 0.05). These results suggest that the variability of endpoint force varied in a direction-specific manner in E group, whereas a constant variability existed in all directions in E group.

**Figure 3:**
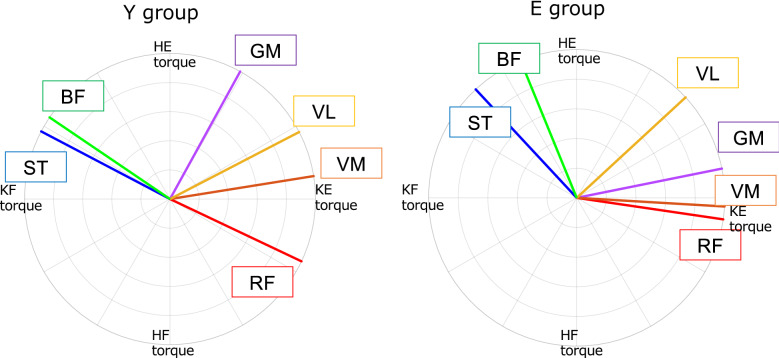
Circular plots of Y group (left) and E group (right) showing the preferred direction of each tested muscle. BF, biceps femoris; GM, gluteus maximus; RF, rectus femoris; ST, semitendinosus; VL, vastus lateralis; VM, vastus medialis in torque plane; KE, knee extension; KF, knee flexion; HE, hip extension; HF, hip flexion.

Figure 3 shows the PD values for each group; GM, VM, and VL were in the first quadrant in both groups. The ST and BF groups had PDs in the second quadrant. Additionally, the RF had PD in the fourth quadrant. Between Y group and E group, there were significant differences in GM and RF (GM: *P* < 0.001, *P* < 0.001) (Table 1).

**Table 1:** Comparison of preferred direction (PD) in each group.

	Y group	E group	
GM	57.0 ± 29.6°	11.4 ± 16.9°	*P* < 0.001
RF	326.8 ± 27.0°	351.7 ± 20.6°	*P* < 0.001
VM	10.0 ± 18.3°	3.4 ± 29.1°	
VL	26.0 ± 12.6°	42.7 ± 17.5°	
ST	154.9 ± 20.0°	133.0 ± 41.4°	
BF	149.0 ± 30.0°	112.3 ± 43.0°	

*GM* gluteus maximus, *RF* rectus femoris, *VM* vastus medialis, *VL* vastus lateralis, *ST* semitendinosus, *BF* biceps femoris.

**Figure 4:**
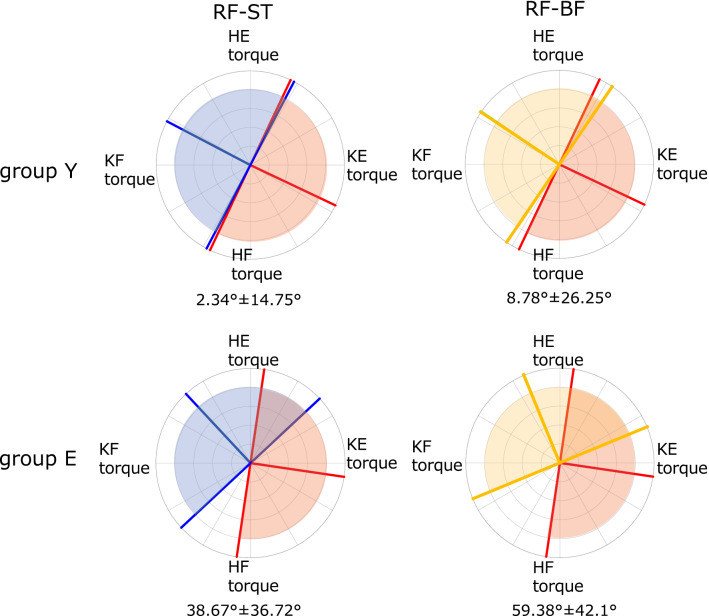
Circular plots of Y group (top) and E group (bottom) showing the range of overlap of the muscle’s preferred direction (PD) between rectus femoris (RF) in red and the hamstrings. The semitendinosus (ST) is shown in blue (left), while the biceps femoris (BF) in yellow (right) in the torque plane. KE, knee extension; KF, knee flexion; HE, hip extension; HF, hip flexion. The lines represent the PD and PD ± 90° of the tested muscle, and the matching colors represent the range. The values at the bottom of each graph represent the mean and standard deviation of the angles of overlapping ranges for each PD ± 90°.

**Figure 5:**
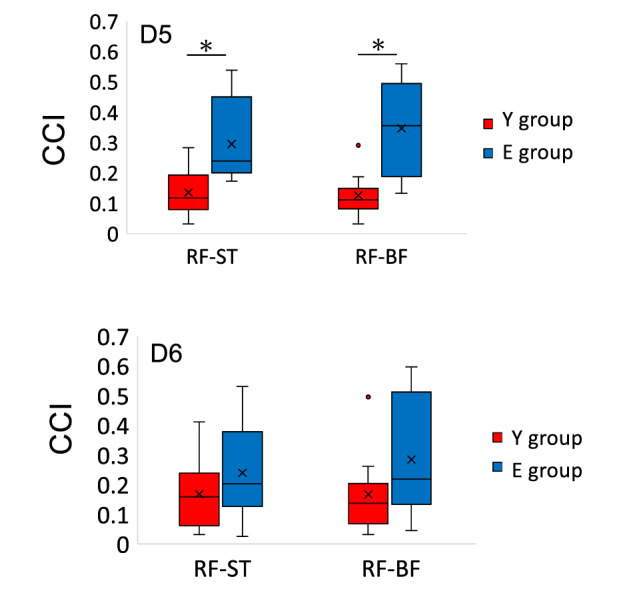
Box plot comparing the co-contraction index (CCI) for directions D5 and D6 in the torque plane of the muscle groups, rectus femoris-semitendinosus (RF-ST) and RF-biceps femoris (BF), in Y group and E group. The bold line in the box indicates the median. The lower and upper ends of the box indicate the first and third quartiles, respectively, while the lower and upper ends of the bars are the minimum and maximum values, respectively. Single dots represent interquartile range outliers. The asterisks represent significantly higher values in E group than Y group.

Figure 4 shows the overlapping ranges of RF-ST and RF-BF. In both cases, E group had a significantly wider overlap range than Y group (RF-ST: *P* = 0.0295, RF-BF: 0.0185). Furthermore, the overlapping area covered the first quadrant. The CCI of the RF and hamstrings (ST and BF, respectively) at D5 and D6, which are included in the overlapping range, was calculated; D5 had a significantly higher CCI in RF-ST and RF-BF in E group than in Y group (RF-ST: *P* = 0.0087, RF-BF: *P* = 0.0013). No significant differences were found at D6 (Fig. 5). These results suggest that E group has more co-activation between RF and hamstrings (ST and BF) in the generation of hip and knee extension torque (first quadrant) compared to Y group.

**Figure 6:**
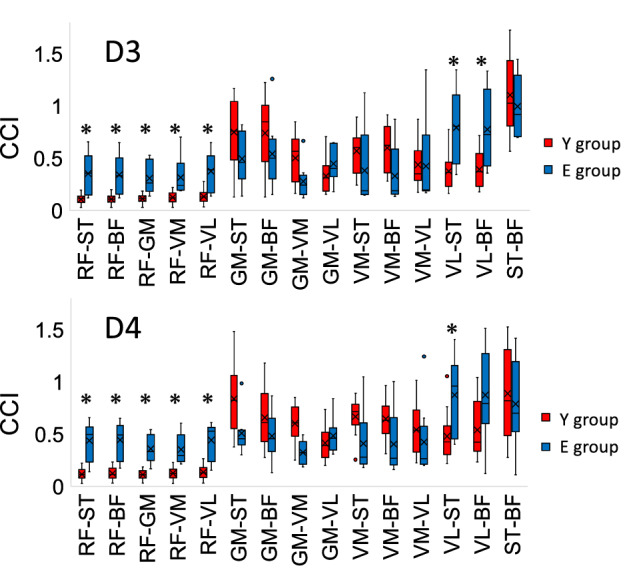
Box plot comparing the co-contraction index (CCI) for directions D3 and D4 between the torque plane of all tested muscle groups and the treatment Y group and E group. The bold line in the box indicates the median. The lower and upper ends of the box indicate the first and third quartiles, respectively, while the lower and upper ends of the bars represent the minimum and maximum values, respectively. Single dots represent interquartile range outliers. Asterisks represent significantly higher values in E group than Y group. BF, biceps femoris; GM, gluteus maximus; RF, rectus femoris; ST, semitendinosus; VL, vastus lateralis; VM, vastus medialis.

**Figure 7:**
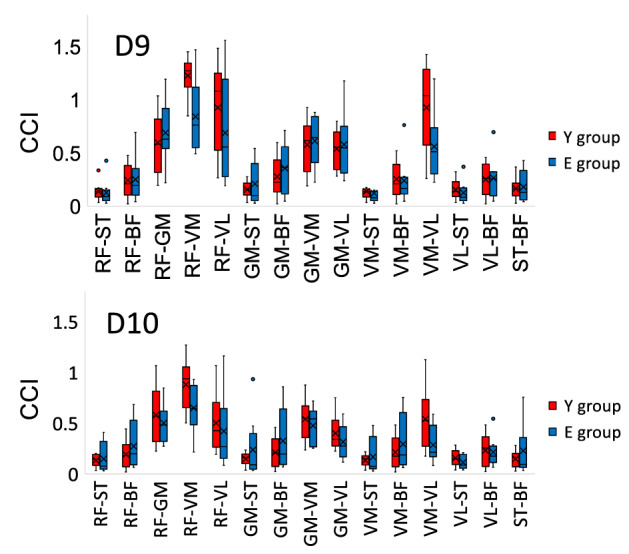
Box plot comparing the co-contraction index (CCI) for directions D9 and D10 in the torque plane of all tested muscle groups in the treatment Y group and E group. The bold line in the box indicates the median. The lower and upper ends of the box indicate the first and third quartiles, respectively, and the lower and upper ends of the bars represent the minimum and maximum values, respectively. Single dots represent interquartile range outliers. BF, biceps femoris; GM, gluteus maximus; RF, rectus femoris; ST, semitendinosus; VL, vastus lateralis; VM, vastus medialis.

The results of the CCI are shown in Figs. 6 and 7. In this study, we focused on D3, 4, 9, and 10, which were found to have a significantly low η in E group (Fig. 2). A group comparison of CCI between each muscle in directions D3 and D4 is shown in Fig. 6. At D3, E group had a significantly higher CCI in RF-ST, RF-BF, RF-GM, RF-VM, RF-VL, VL-ST, and VL-BF than Y group (*P* < 0.05). At D4, E group had a significantly higher CCI in RF-ST, RF-BF, RF-GM, RF-VM, RF-VL, and VL-ST than Y group (*P* < 0.05). A group comparison of CCI between each muscle in directions D9 and D10 is shown in Fig. 7. No significant differences were observed in the CCI between the muscles at D9 and D10. These results indicate that E group has a high muscle co-activation at D3 and D4 (second quadrant).

## Discussion

This study, we investigated the coordinated activity patterns of muscles based on cosine tuning change in the elderly during an isometric force exertion task. We also clarified whether these coordinated activity patterns contribute to the control of hip and knee joint torque and endpoint force as co-activation. The results suggest that co-activation between RF and ST/BF increases with changes in muscle PD in the elderly. In addition, the η values were significantly low, suggesting that the co-activation of multiple muscles may contribute to endpoint force exertion.

The PD values were in the same quadrant in both groups (Fig. 3), which is consistent with previous studies. For example, Nozaki et al.^17^ reported that the PD of each muscle is located in the first quadrant for the GM, VM, and VL, the second quadrant for the ST and BF, and the fourth quadrant for the RF. Moreover, Hagio et al.^20^ showed that the endpoint force covariance exhibited an ellipsoid pattern with higher values in the first and third quadrants of the force plane.

Regarding cosine tuning, the relationship between the η values and PD can be considered as follows: PD range (RF, ST, and BF) for the bi-articular muscles was consistent with a higher range of covariance for the η values (Figs. 2 and 3). An endpoint covariance is high when only a few muscles highly contribute to the generation of endpoint force in that direction^20^. In a skillful movement, healthy young individuals produce net torque at a joint by optimally scaling the activation of the prime movers and concurrent activity of the antagonist muscles^4^. Kutch et al.^22^ reported that the covariance between each muscle PD and endpoint force matched in an isometric force exertion task for the fingertips. In this study, the ST, BF, and RF PDs responsible for both joint torque generation were in the second (hip extension and knee flexion torque) and fourth quadrants (hip flexion and knee extension torque). Therefore, these muscles, as prime movers, might greatly contribute to endpoint force generation, thus leading to high covariance.

In contrast, E group had significantly lower η values in the second and fourth quadrants than Y group. Older adults generate the desired torque with a different neural strategy that involves a near complete activation of the agonist muscles combined with a disproportionately elevated co-activation of the antagonist muscles^4^. Additionally, co-activation of the antagonist muscle is known to cause fluctuations in the motor output response^32^. Here, all muscle PDs of E group were in the same quadrant as those in Y group. However, the PDs of the RF significantly shifted toward the first quadrant. Therefore, this might have affected the range of antagonist muscles in E group. The hamstrings were highly co-active with the RF in the second quadrant at D3 and D4 where they showed a high contribution as prime movers (Fig. 6). The agonist and antagonist muscles during voluntary movements are coordinated by disynaptic reciprocal Ia inhibition in the spinal cord^33^. Therefore, when the agonist muscle is active, the antagonist muscle is inhibited. In contrast, older adults experience a decrease in reciprocal reflex inhibition with age, which increases the amount of antagonist muscle activity observed during voluntary movements^4^. This suggests that age-related changes in the muscle control mechanism might generate higher co-activity of the hamstrings and RF at D3 and D4.

In Y group, the η value in the first quadrant was significantly low (Fig. 1). Furthermore, the PDs of GM, the monoarticular muscle of the hip joint, and VM and VL, the monoarticular muscles of the knee joint, were located in the first quadrant (Fig. 3). This may reflect the coordinated activity of each muscle with different PDs^22^. The musculoskeletal system of the lower extremity does not comprise bi-articular muscles that generate hip and knee joint extension torques. Furthermore, joint torque generation requires simultaneous muscle contraction of each monoarticular muscle. Therefore, these muscles might have been co-activated and contributed to the endpoint force generation. These results indicate that in the human lower-limb musculoskeletal system, which lacks bi-articular muscles that generate hip and knee joint extension torques, each muscle with different PD contributes to endpoint force generation, while mobilizing in a coordinated manner to generate hip and knee joint torques in the first quadrant.

The η value in the first quadrant in E group was not significantly different from that in Y group (D5 and 6 in Fig. 2); hence, the endpoint force generation by the mechanism described earlier was similar in both groups. Nonetheless, a mechanism of multiple muscles coordinated activity specific to E group was also observed. In E group the PD of the RF significantly shifted toward the first quadrant (Table 1). Furthermore, the overlapping range of RF-ST and RF-BF co-activity was significantly wider in E group than in Y group (Fig. 4). The CCI, which represents the level of co-activity between each muscle, showed a significantly higher co-activity in E group in RF-ST and RF-BF at D5 in the first quadrant (Fig. 5).

These results suggest that E group regulates hip and knee joint extension torques of various magnitude combinations, co-activating the RF and hamstrings. Additionally, D5 in the torque plane corresponds to the endpoint force generation in the vertical, downward direction when converted to the force plane (Figs. S5 and S6). Thus, it is possible that E group was co-activating the muscles more than Y group during force control to direct the endpoints vertically downward, as well as during the control of hip and knee joint extension torques. Hogan et al. suggested that stiffness control or independent position/force control can occur in the presence of an antagonistic pair of bi-articular muscles^34,35^. Moreover, the co-activation of an antagonistic pair of bi-articular muscles contributes to endpoint force control in terms of mechanical control engineering^14^. Indeed, Hoff et al. reported that antagonistic biarticular muscles play an important role in directing the endpoint force of the lower limb vertically to the floor^36^. This suggests that the antagonistic pair of bi-articular muscles, the RF and hamstrings, must contract together to exert a vertical, downward force. In fact, the RF and hamstrings are active during the phase in which the center of the body mass is lifted upward during sit-to-stand movements^15^. This suggests that the RF and hamstrings co-activation is necessary for the endpoint force exertion at D5 observed in our results. Nevertheless, the results of this study indicate that E group has greater co-activity than Y group. Originally, the hamstrings generated hip extension and knee flexion torque. However, a closed kinetic chain condition causes extension of the knee joint^37^; therefore, conceivably the hamstrings could function as co-activators in knee extension torque generation. A decrease in force production capability, particularly at the knee joint, is a primary alteration in aging^38,39^. This may result in a mechanical output unable to achieve the desired force exertion level^3^. Motor unit activation may also become ineffective with advanced age due to an altered balance between the excitation and inhibition of spinal circuits^5^. In these cases, the mobilized muscle activity pattern may differ from that of young people, although they exert the same force. Therefore, it is possible that the hamstrings are mobilized as coactivator muscles to generate knee extension torque.

The RF and hamstrings are co-activated in the elderly during gait tasks^9^. This co-activity strategically enhances joint stability^13^. Most studies reporting co-activation in the elderly have been performed on tasks that impose mechanical loading on the knee joint, such as gait^40,41^, while herein, the task was conducted in a non-weight-bearing fashion to exclude mechanical effects. Nevertheless, the results of force covariance and muscle coordinated activity patterns showed an increase in co-activation of the muscles in E group. This co-activation differs from strategies meant to increase joint stiffness for movement in any direction, as it is difficult to exert a force in the desired direction when the joints are excessively stiffened. Additionally, based on a previous study, the subjects in this study were instructed not to stiffen their leg excessively. Nozaki et al.^17^ showed no joint-stiffening co-activity. Hence, the co-activation in our results was also considered to be a co-activation for joint torque and endpoint force exertion, rather than a strategy to increase joint stabilization. Our results indicate that the mechanism of muscle cooperative activity determined by the cosine tuning of each muscle PD may affect hip and knee joint torque generation and endpoint force exertion.

### Limitations

The study was conducted with the participant in a non-weight-bearing, side-lying position with the measured lower extremity suspended by a sling. Under loading conditions, muscle activity is essential for joint stability. Therefore, dynamic tasks such as walking and standing, in which the mechanical environment is different, cannot be treated the same as in the present study. The following issues must be resolved for future applications in dynamic tasks: first, changes in PD due to changes in joint angles must be considered. Since the PD is affected by mechanical direction (MD), it is expected that joint angle changes resulting in different MDs would exhibit different PD distributions. The first task was performed to only determine the distribution of PD for all combinations of hip and knee joint angles during walking and standing.

Second, an appropriate task must be selected under loading conditions. Moreover, to accurately assess PD, various strengths and combinations of joint torque exertion must be applied to the two joints of interest. Thus, PD could not be accurately obtained from the dynamic task itself, which is constrained to a certain strength and combination of torque exertion. Consequently, it is necessary to examine a dynamic task in which various torque exertions can be imposed under loading conditions and to verify the PD in such a task. Subsequently, using this PD, the contribution of each muscle to the torque exertion obtained in tasks such as walking and standing should be calculated, and the PD should be compared with the PD of the muscle activity measured in the actual task. Future experiments should investigate the effectiveness of cosine tuning in dynamic tasks by verifying the similarity between estimated muscle activity obtained from the PD and the muscle activity measured in tasks such as walking and standing.

The possibility that the deviation of PD in the elderly was a result of the different MDs of each muscle was considered. Nozaki et al.^17^ calculated the MDs of healthy participants by referring to the value of Delp^42^, which is specific for young adults and cannot be directly applied to the elderly. Additionally, if the arm length varies with differences in muscle thickness in the elderly, it is necessary to use magnetic resonance imaging, computed tomography, and ultrasound to calculate the MD in detail. Here, the MD could not be calculated since these evaluations were not conducted. Future studies should investigate the quantification of MD in the elderly population and its relationship with PD deviation, which will provide novel insights into the cooperative activity patterns of multiple muscles in the elderly.

Finally, it should be noted that these results are for males only. Owing to sex differences in muscle strength and muscle mass, MD may differ between males and females. In the future, it will be necessary to quantitatively evaluate sex differences in muscle activity patterns.

## Conclusion

To summarize, the co-activation of each muscle PD changes with age; in the elderly this may lead to the use of more muscle co-activation to control torque and force. We demonstrated that co-activation in the elderly is not only a stabilizer of unstable joints, but also a muscle control strategy for muscle cooperative activity. Research on age-specific mechanisms is important to design personalized therapeutic strategies for different populations of patients.

## Supplementary Information

Supplementary Information.

## Data Availability

All data generated or analyzed during this study are included in this published article [and its supplementary information files].
