# Supplementary material for: Muscle co-activation in the elderly contributes to control of hip and knee joint torque and endpoint force
Source: Sci Rep. 2023 May 2;13:7139. doi: 10.1038/s41598-023-34208-6 (PMC10154344; doi:10.1038/s41598-023-34208-6)
Supplement: Supplementary file 1 — Supplementary Information. [file 41598_2023_34208_MOESM1_ESM.pdf]

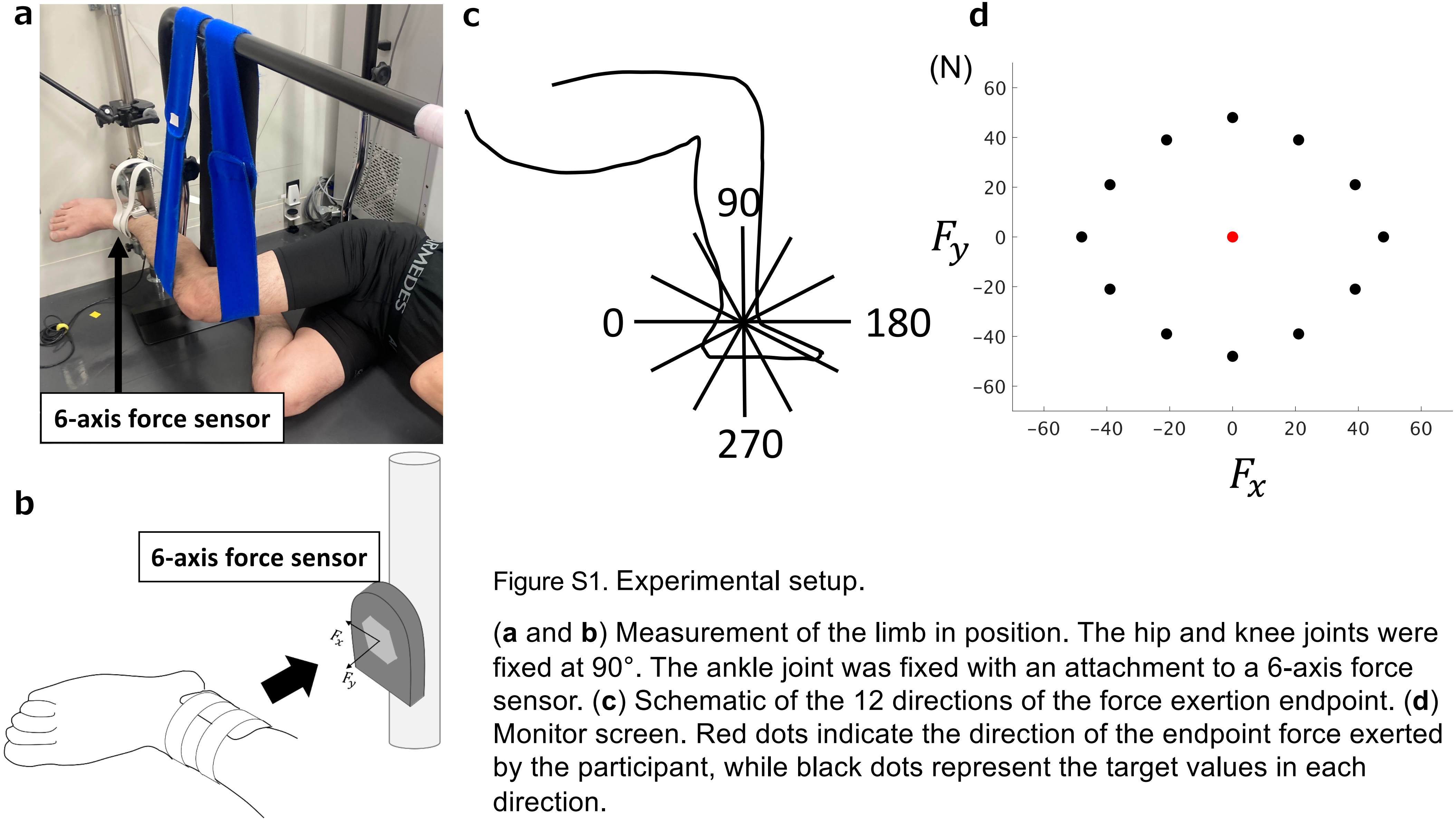

Figure S1. Experimental setup.

(**a** and **b**) Measurement of the limb in position. The hip and knee joints were fixed at 90°. The ankle joint was fixed with an attachment to a 6-axis force sensor. (**c**) Schematic of the 12 directions of the force exertion endpoint. (**d**) Monitor screen. Red dots indicate the direction of the endpoint force exerted by the participant, while black dots represent the target values in each direction.

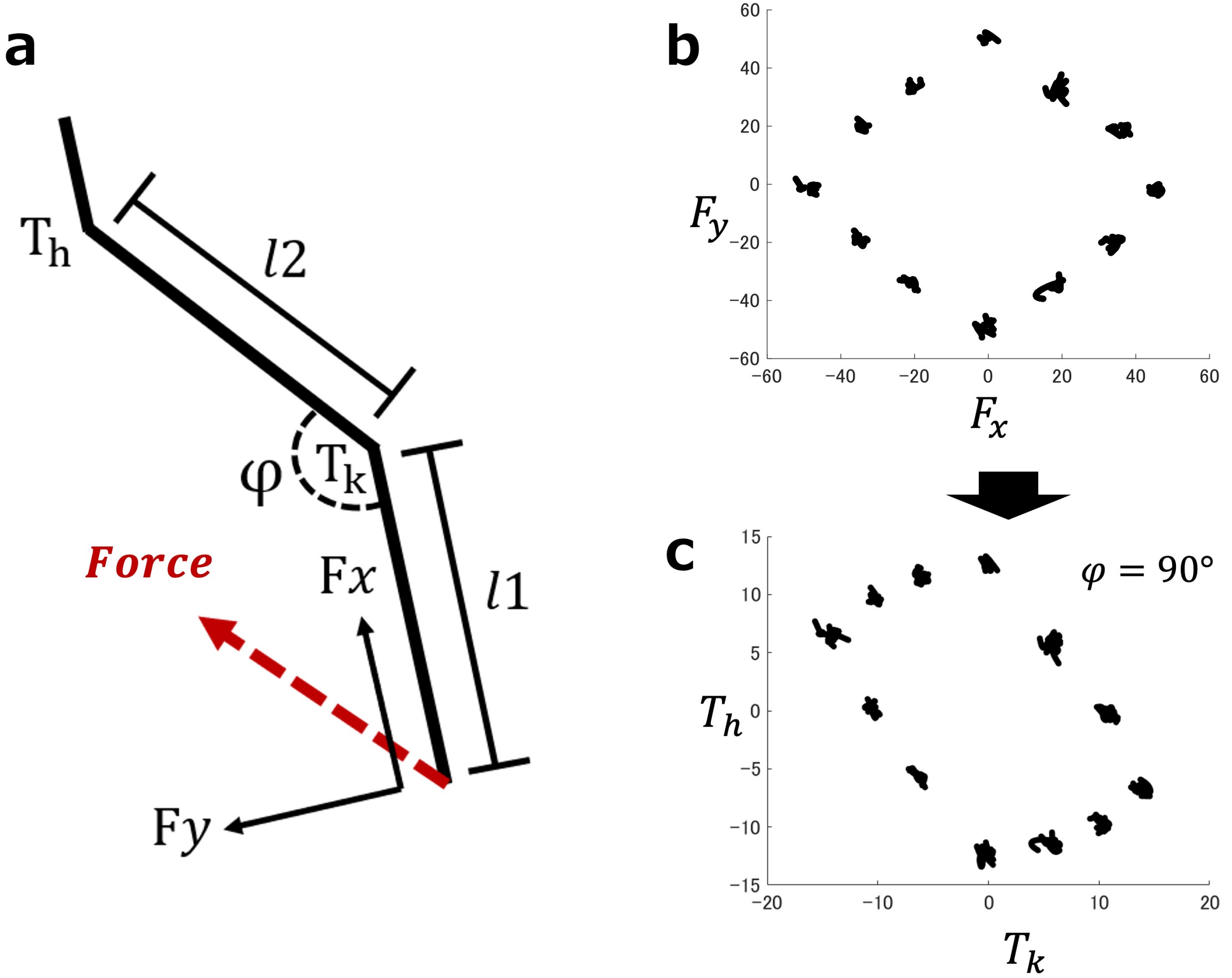

Figure S2. Conversion of the force vectors $F_{(x,y)}$ to torque data using equation (1).

(**a**) Schematic illustration of parameter definition. (**b**) Graph showing the force plane. (**c**) Graph showing the torque plane after conversion.

l_1, length of the thigh; l_2, length of the shank; φ, angle of the knee joint; T_h, hip joint torque; T_k, knee joint torque

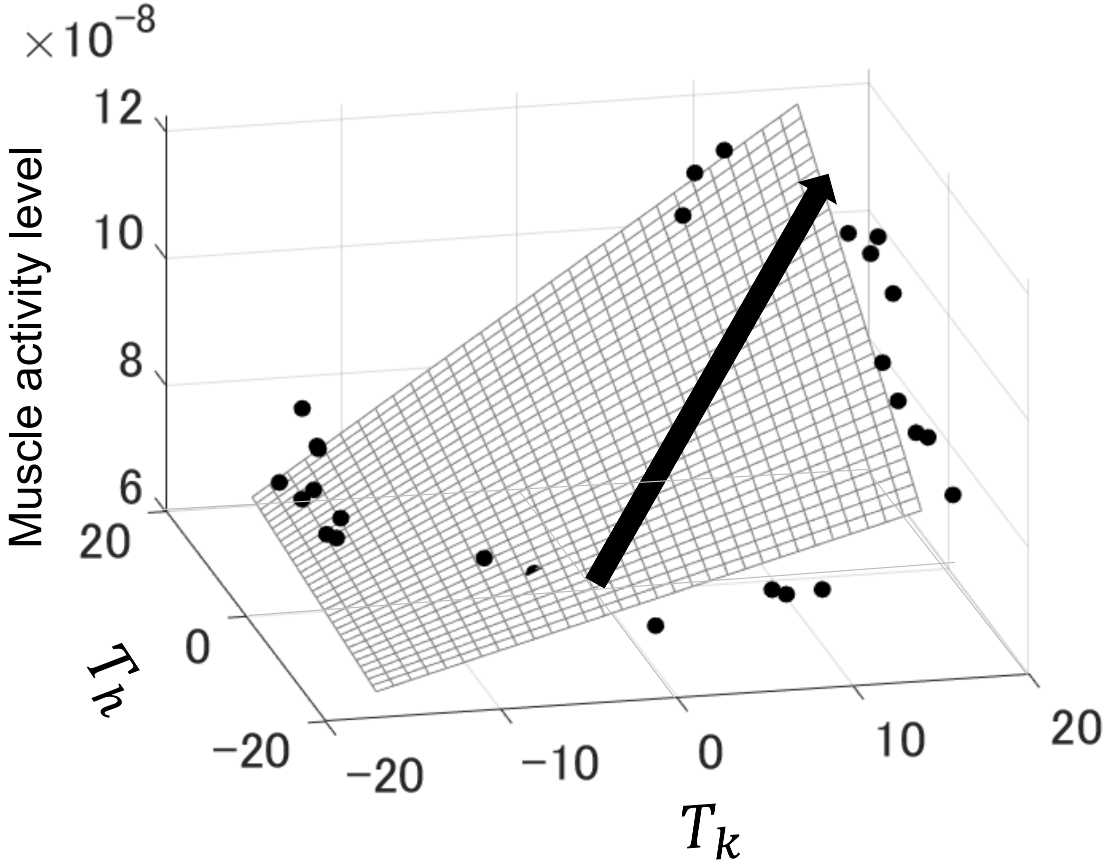

Figure S3. Multiple linear regression analysis three-dimensional graph showing the distribution of the muscle activity levels on the torque plane.

The arrow indicates the preferred direction (PD).

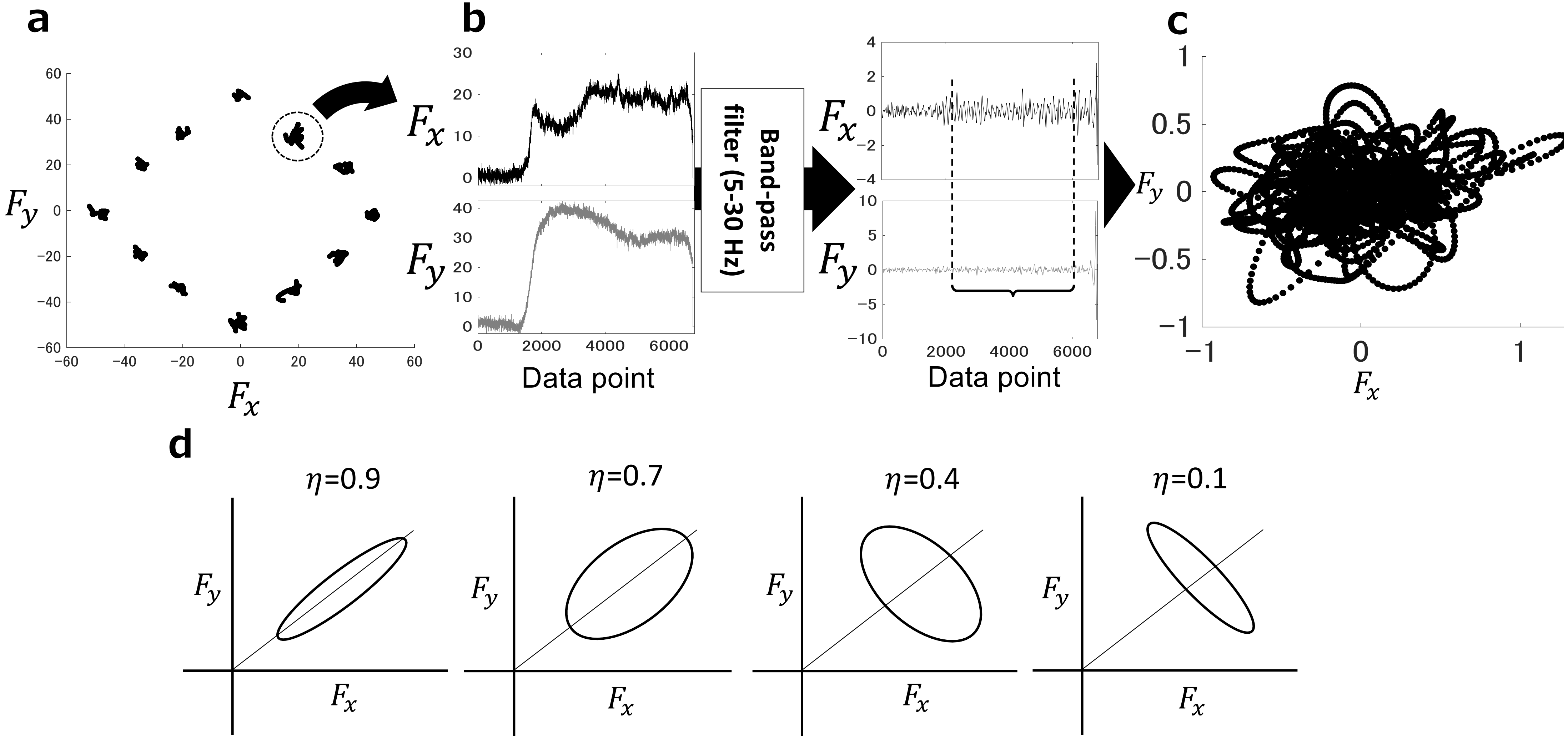

Figure S4. Procedure for calculating the covariance of the endpoint force (η) [22].

(**a**) Selection of a single endpoint force in the force plane. (**b**) The endpoint force vectors F_x and F_y were filtered with a bandpass filter (5–30 Hz). Dashed lines show the analyzed range (at 4 s) of each force. (**c**) Two-dimensional scatter plot of the filtered vectors F_x and F_y (4,000 data points). (**d**) Some examples of the geometry of (**c**) at values of η. A higher value of η implies greater covariance between F_x and F_y.

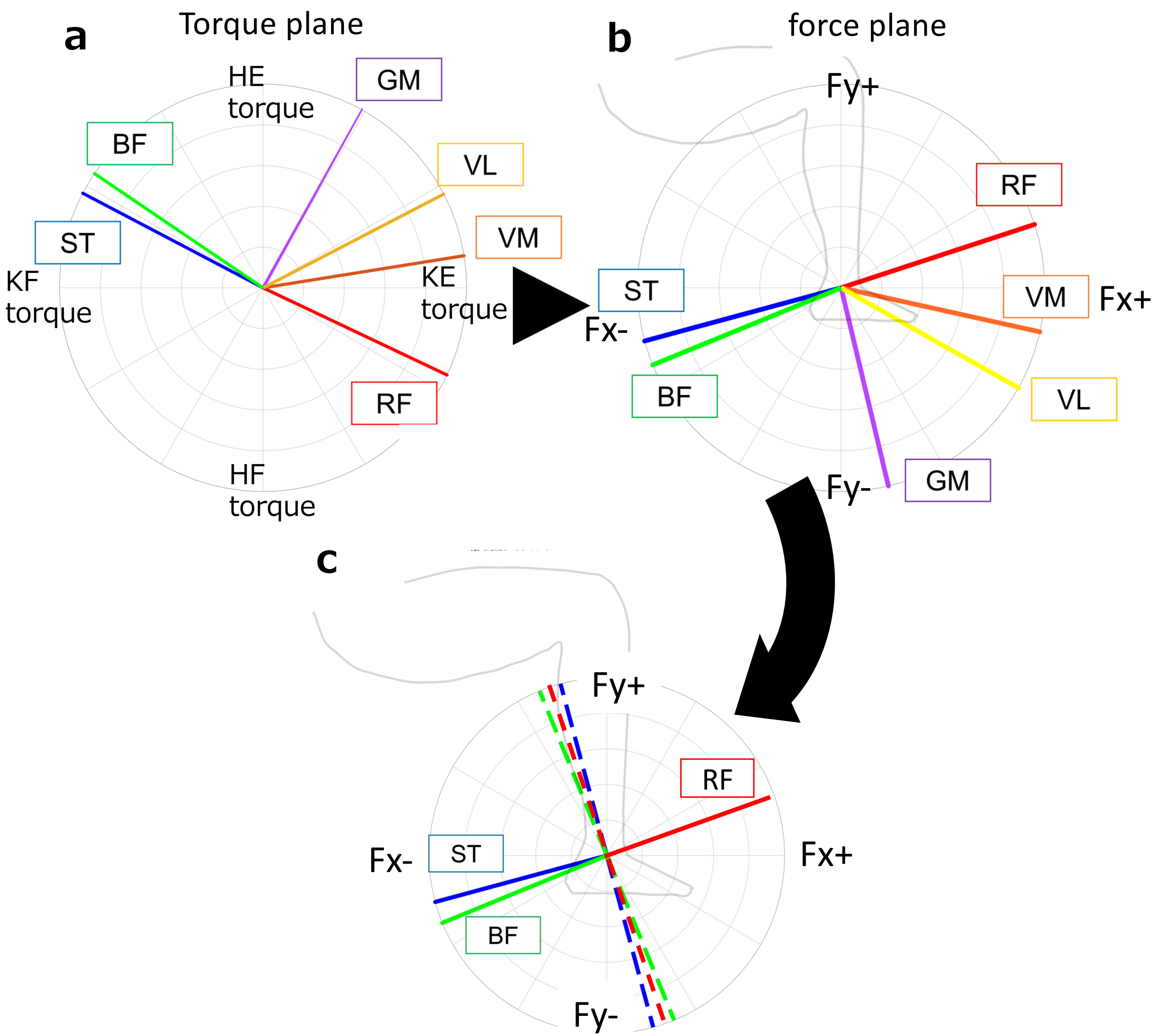

Figure S5. Transformation of the preferred direction (PD) from the torque plane to the force plane of the Y group.

(**a**) Each muscle's PD in the torque plane. (**b** and **c**) Endpoint forces of each muscle's PD in the force plane. (**c**) Isolation of RF, ST, and BF, showing the PD (solid line) and the PD ± 90° (dashed line).

BF, biceps femoris; GM, gluteus maximus; KE, knee extension; KF, knee flexion; HE, hip extension; HF, hip flexion; RF, rectus femoris; ST, semitendinosus; VL, vastus lateralis; VM, vastus medialis

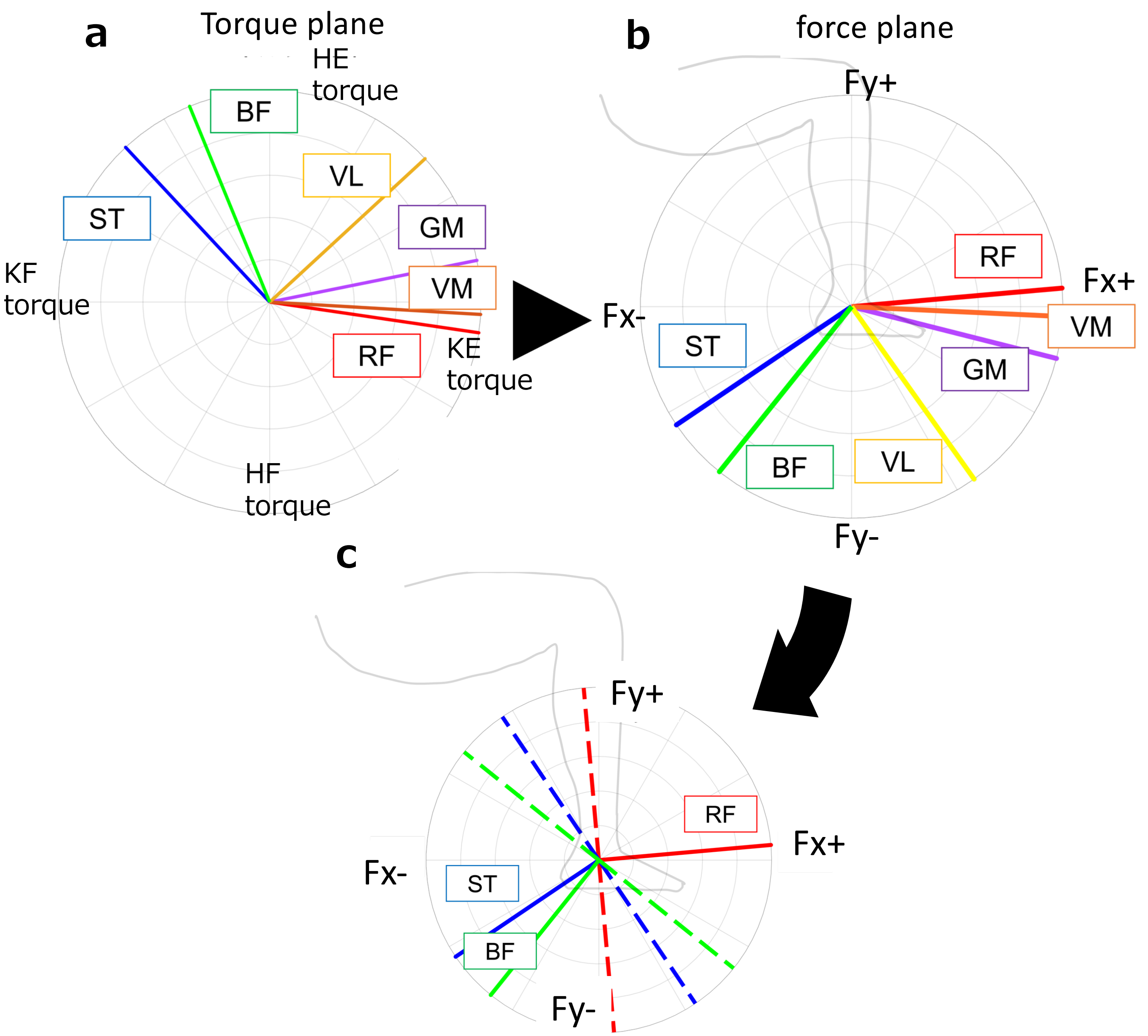

Figure S6. Transformation of the preferred direction (PD) from the torque plane to the force plane of the E group.

(**a**) Each muscle's PD in the torque plane. (**b** and **c**) Endpoint forces of each muscle's PD in the force plane. (**c**) Isolation of RF, ST, and BF, showing the PD (solid line) and the PD ± 90° (dashed line).

BF, biceps femoris; GM, gluteus maximus; RF, rectus femoris; ST, semitendinosus; VL, vastus lateralis; VM, vastus medialis
